# Breaking the Itch–Scratch Cycle: Topical Options for the Management of Chronic Cutaneous Itch in Atopic Dermatitis

**DOI:** 10.3390/medicines6030076

**Published:** 2019-07-18

**Authors:** Ian P. Harrison, Fabrizio Spada

**Affiliations:** Department of Research and Development, Ego Pharmaceuticals Pty Ltd., 21-31 Malcolm Road, Braeside VIC 3195, Australia

**Keywords:** chronic pruritus, skin, atopic dermatitis, ceramide, pine tar

## Abstract

Chronic itch is an unpleasant sensation that triggers a desire to scratch that lasts for six weeks or more. It is a major diagnostic symptom of myriad diseases, including atopic dermatitis for which it is the most prominent feature. Chronic itch can be hugely debilitating for the sufferer, damaging in terms of both the monetary cost of treatment and its socioeconomic effects, and few treatment options exist that can adequately control it. Corticosteroids remain the first line treatment strategy for atopic dermatitis, but due to the risks associated with long-term use of corticosteroids, and the drawbacks of other topical options such as topical calcineurin inhibitors and capsaicin, topical options for itch management that are efficacious and can be used indefinitely are needed. In this review, we detail the pathophysiology of chronic pruritus, its key features, and the disease most commonly associated with it. We also assess the role of the skin and its components in maintaining a healthy barrier function, thus reducing dryness and the itch sensation. Lastly, we briefly detail examples of topical options for the management of chronic pruritus that can be used indefinitely, overcoming the risk associated with long-term use of corticosteroids.

## 1. Introduction: the Pathophysiology of Pruritus

Itch, formally “pruritus” from the Latin “prurit” (“to itch”), has been defined as “an unpleasant cutaneous sensation which provokes the desire to scratch” [1]. It is a common response to many stimuli, and a feature of many diseases, from systemic conditions such as renal insufficiency [2] to skin diseases such as atopic dermatitis (AD), of which itch is *the* major symptom [3,4]. Unlike acute itch, which is transient and usually overcome quickly by scratching the affected area, chronic itch is persistent and debilitating, to the point that the act of scratching can actually aggravate the problem even while providing relief [5]. While there are many treatment options available for chronic cutaneous pruritus, most are unsatisfactory due to the complex nature of the itch response and the subjective nature of the problem itself. The mere thought of itching can confound treatment outcomes: mentioning itch to a person will usually elicit an itch response in them that could damage the skin further, even if the treatment is successfully dealing with the underlying condition. Indeed, the effect of psychology can make it extremely difficult to assess the efficacy of antipruritic agents: it has been noted that the placebo effect can be as high as 50% in pruritus patients [6]. Successful management strategies ideally should incorporate into the chosen treatment regimen the daily use of easily-accessible, efficacious topical preparations designed primarily to tackle the itch. The benefits of these products could conceivably be twofold: physically alleviating the itch stimulus while helping to overcome the psychological need to scratch by allowing for the *ad libitum* application of the product. This review aims to provide a brief overview of itch, its impact in atopic dermatitis, and detail the components of the stratum corneum that play an integral part in maintaining barrier function and, in doing so, help to manage the itch. We also briefly detail current topical options designed specifically for the management of itch.

### 1.1. Classifying Itch

Itch can be broadly classified into four distinct clinical categories: neurogenic, neuropathic, psychogenic and pruritoceptive [7]. Experiencing itch from one of these categories does not preclude the sufferer from experiencing an additional one or more other categories of itch concurrently. Neurogenic itch stems from disorders affecting organ systems, such as renal failure [8] and liver disease [9], while neuropathic itch can be a result of lesions on or pathological changes to the afferent pathway signaling to the central nervous system [10]. Psychogenic itch refers to itch associated with psychological maladies that do not have an underlying physiological etiology, such as delusional parasitosis [10]. The most common category of itch, and the category of note to this review, is pruritoceptive or cutaneous itch, an itch caused by inflammation of the skin [11]. This inflammation can be localized and transient as a result of, say, an insect bite, or it can be chronic and widespread as a result of disease. The fact that cutaneous pruritus is so common can be attributed to the nature of the skin itself: as the body’s barrier to the external environment, the skin is subject to the effects of both endogenous mediators of itch (inflammation for example) and exogenous allergens, irritants and mechanical disruption.

### 1.2. Neural Mechanisms of Itch

The exact mechanisms of itch are poorly understood, such is its complexity. Numerous theories exist to try to explain pruriceptive sensation, notably the specificity and the pattern theories. The specificity theory posits that there are specific nerve fibers and neurons that transmit the itch response to the central nervous system (CNS), whereas the pattern theory suggests that itch is encoded across numerous sensory receptors and neurons, and that the pattern of this neuronal activity is what determines the sensation that is experienced [12,13]. Currently, the literature seems to favor the specificity theory [14].

**Figure 1:**
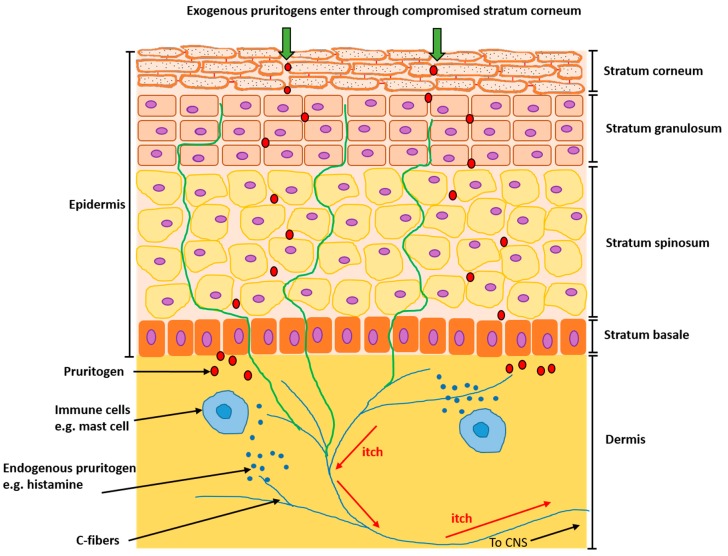
The itch pathway. Exogenous itch-causing substances, pruritogens, enter through the compromised stratum corneum and journey through the layers of the skin until they bind to their receptors on sensory afferent nerves, or C-fibers (in blue), triggering a signal which travels up the central nervous system (CNS) to the brain, where it is recognized as an itch. In addition, endogenous pruritogens such as histamine can be produced by cells of the body, such as mast cells. Nerve distribution and density is increased within the epidermis in AD skin (in green).

The perception of itch starts when an itch-causing substance, or pruritogen, enters through the stratum corneum and binds to its receptors on sensory afferent nerves, or C-fibers, which transmit the resulting signal to the CNS where the brain interprets it as an itch and initiates a scratch response. Endogenous pruritogens can also be produced by both keratinocytes and immune cells such as mast cells, which produce histamine, a key mediator of itch (Figure 1). The distribution, thickness and density of intraepidermal nerve fibers is much higher in AD skin, which may exacerbate the itch response [15].

### 1.3. Endogenous Mediators of Pruritus

In addition to exogenous mediators of pruritus, numerous endogenous biological mediators exist that can elicit a pruritoceptive response in the skin. First and foremost is histamine, the most commonly used experimental pruritogen. Histamine is primarily produced by dermal mast cells in response to allergic stimuli, hence the first-line use of antihistamines as treatments for allergies. Histamine binds four known receptors, H1 to H4, with H1 being the primary receptor subtype responsible for itch. These receptors are located on sensory neurons, which transmit the signal created by the activated receptors to the brain to be recognized as itch. Crucially for AD, however, is that it is non-histaminergic itch pathways that are thought to predominate in the disease. A 2017 study of patients with AD found that itch sensation to cowhage, a tropical legume native to Asia and Africa and a potent pruritogen, was significantly greater both intra- and extralesionally than control compared with itch responses to histamine, which were not significantly different in AD skin versus control skin [16]. The monoamine neurotransmitter serotonin and the vasodilator bradykinin, while both relatively non-pruritogenic in normal skin, elicit strong pruritic responses in AD skin that are also histamine-independent [17]. Endogenous serine proteases such as tryptase are known to elicit an itch response, and are also upregulated in AD skin [18,19]. Interleukin-31 (Il-31), a Th2 cytokine of the IL-6 family of cytokines, is another prominent endogenous mediator of pruritus, especially in AD skin. Elevated levels of IL-31 have been observed in AD skin [20], and IL-31 receptor A expression is also most abundant in the dorsal root ganglia, the primary site of cutaneous sensory neurons [20]. A 2018 meta-analysis of studies looking at Il-31 in AD found that serum levels of IL-31 are proportional to the severity of AD, with the greatest levels of serum IL-31 in patients with severe AD [21]. 

## 2. The Stratum Corneum, Atopic Dermatitis and Pruritus

**Figure 2:**
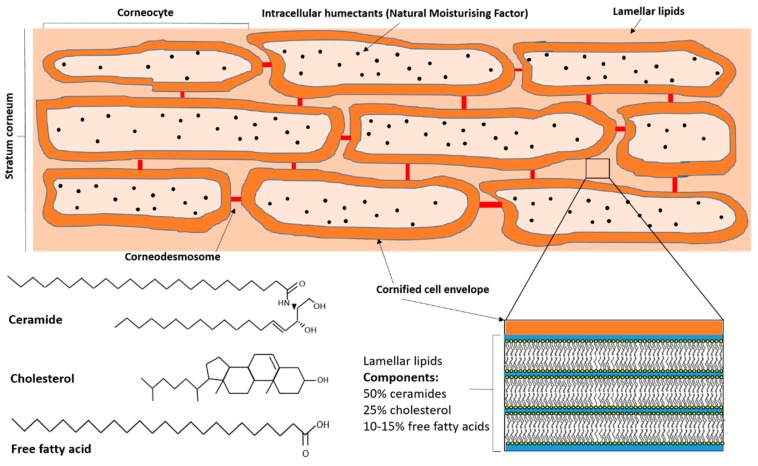
Schematic representation of the stratum corneum, with a view of the composition of the lamellar lipid layer. The typical chemical compositions of the major skin barrier lipids (ceramides, cholesterol and free fatty acids) are shown at bottom left.

The stratum corneum, the outermost layer of the skin, forms the protective barrier between the inner body and the outside world [22]. Its barrier functions are numerous, from the prevention of trans-epidermal water loss from the epidermis to the external environment, to protection from external pathogens. It is composed of approximately 15–25 layers of dead, flattened keratinocytes (corneocytes) embedded in a lipid bilayer [23], which gives rise to the “brick and mortar” model most commonly used to describe it [24]. The lipid bilayer consists of approximately 50% ceramides, 25% cholesterol and 10–15% free fatty acids, with small amounts of glucosylceramides and phospholipids [24]. Instead of the plasma membrane that encases living cells, corneocytes are surrounded by an insoluble cornified cell envelope composed of a monolayer of ceramides, and are held together in the lipid bilayer by corneodesmosomes, modified desmosomes from the uppermost layer of the stratum granulosum [25] (Figure 2). 

AD, the most common chronic inflammatory skin disease, affects up to 3% of adults and up to 20% of children worldwide [26], with its incidence increasing in developing countries [27]. While the pathophysiology of AD is complex and not entirely understood, it is universally acknowledged that an essential symptom and diagnostic feature of the disease is the intense chronic itch [28]. The itch, combined with the scratching it necessitates, exacerbates the morbidity of the disease and can lead to physical damage: the act of scratching compromises the integrity of the skin, damaging that crucial barrier to the outside world. This damaged barrier essentially becomes an open border, allowing for passage through it from either side: the skin of AD sufferers is extremely dry, owing to the loss of moisture [29], but it also tends to be more susceptible to infection, as external pathogens take advantage of the damaged barrier and compromised immune function [30]. In addition, the dysfunctional barrier seen in AD also results in enhanced antigen penetration, leading to exacerbated allergic reactions. Moisture loss and pathogen infiltration exacerbates dryness and can lead to inflammation, perpetuating the need to scratch, which itself further damages the skin barrier, exacerbates dryness and increases pro-inflammatory mediator release. This sequence of events is commonly referred to as the “itch–scratch cycle” [31,32]. In addition to the physical damage, the itch–scratch cycle can also lead to debilitating psychological sequelae. AD has been associated with depression, anxiety and suicidal ideation, with the severity of these psychiatric diseases proportional to the severity of AD [33]. The intense itch can disrupt sleep, impairing performance at work or school [34], while the altering of early tactile development in infants with AD can negatively impact physical and emotional development [35].

## 3. Topical Options for the Management of Chronic Pruritus

Topical corticosteroids are the recommended first-line treatment option for the management of AD and have been for over half a century [36,37]. Their exact mechanisms of action, like their potency, vary greatly, but all are intended for treatment of steroid-responsive dermatoses such as AD and psoriasis. The least potent, but most widely used, corticosteroid is hydrocortisone. The efficacy of topical corticosteroids in the treatment of AD is well known, but less well known are their benefits specifically in combating the itch associated with AD. A 1988 study by Wahlgren and colleagues developed a method for recording subjective scoring of pruritus in AD patients treated with the potent topical corticosteroid betamethasone dipropionate versus placebo control [38]. They found that itch intensity was significantly lower during corticosteroid treatment, and the onset of the antipruritic effect was rapid, with a statistically significant difference in pruritus between the groups reached within 24 h [38]. A four-week, double-blind randomized clinical trial in 1998 by Maloney et al. compared the weakly potent clobetasol propionate to its vehicle in the treatment of moderate to severe AD [39]. Three symptoms of AD were assessed: pruritus, erythema and induration/papulation. By Day 4, pruritus had significantly improved in patients receiving clobetasol propionate compared with vehicle [39]. Together, these studies show the potential for topical corticosteroids, even weakly potent ones, in the management of chronic itch.

However, topical corticosteroid use does come with risks for the patient. Generally speaking, the more potent the corticosteroid, the greater the risk of adverse effects such as thinning of the skin, folliculitis, impetigo, telangiectasia and atrophy, and, in rarer instances, herpetic infections and adrenal suppression [40,41]. Another drawback of topical corticosteroid use is the potential for tachyphylaxis, a phenomenon whereby continued use of the product results in diminished effects over time [42]. As the products are self-administered, it is conceivable that patients may then start using more of the product in the notion that it will make up for the diminished effects, thereby increasing the likelihood of adverse events. For these reasons, topical corticosteroid use is only ever for short- to medium-term treatment of AD and other similar conditions, an unsatisfactory approach for the management of pruritus, which needs to be persistent. 

Other topical options exist for the treatment of chronic pruritus. Topical calcineurin inhibitors such as tacrolimus and pimecrolimus are frequently used as alternatives to topical corticosteroids for the treatment of AD, yet a 2016 systematic review by Broeders and colleagues found that, while topical calcineurin inhibitors display similar efficacy to topical corticosteroids in treating AD, they were associated with both higher costs and greater adverse events, including burning sensation and pruritus [43]. Due to the importance of histamine in the itch response, topical antihistamines may be of benefit in the treatment of chronic cutaneous itch. However, while topical antihistamines have been found to be effective in treating ocular allergy [44], their effects in treating pruritus of the skin are mixed, and are usually limited in design or inconsistent in findings [45]. Topical doxepin, a potent H1 and H2 receptor antagonist and the only topical antihistamine shown to significantly relieve pruritus in patients with AD, is associated with significant side effects, including allergic contact dermatitis and drowsiness due to systemic absorption [46]. Additionally, due to evidence that the itch in AD is histamine-independent [16], topical antihistamines would have limited to no efficacy in treating AD-related pruritus. Topical capsaicin, or chili pepper extract, has been reported to be an efficacious treatment for itch [47], yet a recent study found that topical capsaicin can actually enhance chronic pruritus, possibly due to the upregulation of Transient Receptor Vanilloid 1-expressing sensory neurons seen in chronic pruritus conditions [48]. Topical anesthetics such as lidocaine and prilocaine are known to have topical anti-pruritic effects by stabilizing sensory fibers and blocking the itch sensation. However, side effects can include allergic contact dermatitis, paresthesia and methemoglobinemia, necessitating the avoidance of these topical agents in children, pregnant women and patients taking oxidizing drugs [49].

Due to the limitations of the treatment options detailed above, there is a need for effective management strategies for chronic itch that encompass topical products that are efficacious but that can also be used indefinitely without the risk of adverse events. One approach would be the use of products designed to support and promote the healthy functioning of the stratum corneum, principally the lipid bilayer and its crucial components: ceramides, cholesterol and free fatty acids. 

## 4. The Key Lipids of the Stratum Corneum and Their Role in Maintaining a Healthy Barrier

### 4.1. The Chemistry of Ceramides, Cholesterol and Free Fatty Acids

Ceramides are simple sphingolipids formed from a combination of two hydrophobic chains: a sphingoid base and a fatty acid [50] (Figure 1). Within the skin, ceramides are synthesized via three different pathways: de novo synthesis via serine palmitoyltransferase in the endoplasmic reticulum, glucosylceramide degradation by β-glucocerebrosidase, and hydrolysis of sphingomyelin by sphingomyelinase [51]. The stratum corneum contains a complex assortment of ceramide subclasses; early studies utilizing thin layer chromatography identified eight ceramide sub-classes [52], but this has since expanded [53,54] to a total of 15 subclasses when 1-O-acylceramides, a new subclass denoted by the very long acyl chains in the N- and O- positions, is included [55]. Ceramide subclasses are differentiated from each other by their individual sphingoid base group (sphingosine, phytosphingosine, dihydrosphingosine, dihydroxy sphinganine or 6-hydroxysphingosine) and their fatty acid chain (alpha-hydroxy acid, non-hydroxy fatty acid or omega hydroxyl fatty acid) [50]. These ceramide subclasses each contain different species based on their specific combination of fatty acids and base groups, with at least 300 [56] and possibly 1000 [57] distinct species present in the stratum corneum. In terms of the chemistry of the different subclasses, the sphingoid base is usually a long chain amino alcohol of approximately 18 carbon molecules [58], while the fatty acid chain can range from about 24 to 38 carbons in length [59]. Cholesterol is the primary sterol in the lipid bilayer of the stratum corneum. Found ubiquitously in all animal tissues, it plays a crucial role in cell membrane integrity and is derived from the oxidation of the hydrocarbon squalene [60]. It consists of a planar four ring nucleus with a flexible side chain (Figure 1). Free fatty acids are usually saturated, straight, long chain compounds (Figure 1). 

### 4.2. The Ceramides of Note in Atopic Skin

While mechanical disruption to the skin barrier caused by scratching the intense itch associated with AD exacerbates the disease, atopic skin is also deficient in numerous stratum corneum components that play a major role in the healthy functioning of the skin barrier, chief among them being ceramides. Not only are ceramide levels significantly reduced in lesional skin of AD sufferers [61], but reduced levels are also seen in nonlesional skin. Ceramide composition is also markedly different in AD skin compared with normal skin [62], and the ratio of ceramides, free fatty acids and cholesterol can have a profound impact on the skin [63]. Chain lengths of ceramides and free fatty acids in AD skin are also shorter than those found in normal skin, leading to increased permeability of the skin barrier [64]. This decrease in ceramide chain length in AD has been strongly associated with skin barrier disruption, increased transepidermal water loss (TEWL) and greater disease severity [64]. The average ceramide chain length in AD skin has been estimated to decrease by 0.64 ± 0.23 total carbon atoms [65].

**Figure 3:**
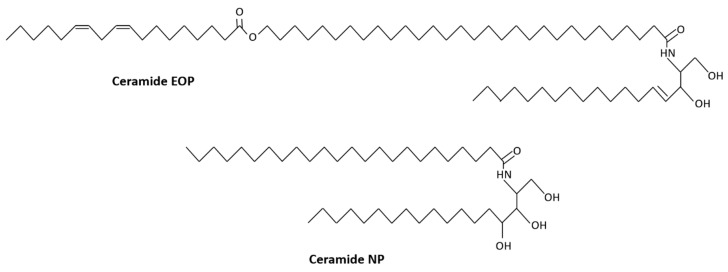
Molecular structures of ceramides EOP and NP. Adapted from [69].

Of the 15 different subclasses of ceramides, ceramides 1 and 3 are most strongly associated with AD. Ceramide 1 (EOP) contains a 30-carbon ester-linked fatty acid acylated to sphingosine, while ceramide 3 (NP) contains a 24-carbon fatty acid acylated to phytosphingosine [66] (Figure 3). Ceramide 1, by way of its long chain length that acts as a connector between the lipid bilayers, plays an important role in the organization of lipids in the stratum corneum [67], while ceramide 3 plays a major role in the morphology of the lipid bilayer [68]. 

A 1998 study by Di Nardo et al. found that levels of ceramides 1 and 3 were significantly lower in AD skin compared with normal skin, while the level of cholesterol was significantly higher [70]. The ratio of ceramides to cholesterol was also significantly lower in AD patients [70]. The decreased levels of ceramides 1 and 3 was correlated with significantly increased TEWL, a major determinant of dry skin. Similarly, Macheleidt and Sandhoff reported in a 2002 study significantly decreased biosynthesis of ceramides 1 and 3 in both lesional and healthy skin of AD sufferers compared with controls, with the authors concluding that these deficiencies may contribute to the increased permeability of the skin barrier in AD [71].

## 5. Topical Ceramide Delivery for Itch Relief

If ceramides, particularly ceramides 1 and 3, are significantly reduced in AD skin, it stands to reason that topical delivery of these ceramides may help to restore the skin’s barrier function and overcome some of the symptoms of AD, specifically itch. Studies in 1993 and 1995 by Man et al. and Yang et al., respectively, found that the topical delivery of lipids accelerated murine barrier repair after tape stripping and disruption by the solvent acetone [72,73]. While not reflective of AD, the fact that the lipid mixture was able to accelerate repair of a compromised barrier would have obvious benefits for the treatment of AD. Subsequent studies refined the lipids and delivery ratio, and applied them to human skin. A 1996 study by Man and colleagues reported that a mixture of ceramides, free fatty acids and cholesterol delivered topically to human skin accelerated barrier recovery, as evidenced by decreased TEWL [74]. This recovery was dependent on carefully calibrated molar ratios of all three lipids; mixtures with only one or two of the lipids, or an incorrect molar ratio, actually impeded barrier recovery [74]. (The importance of a balanced ratio of all three skin lipids when applied to damaged skin has been detailed in a recent review by Elias et al. [75].) A 2002 study by De Paepe et al. showed that the application of a complete mixture of ceramides, cholesterol and free fatty acid significantly improved barrier recovery 14 days after sodium lauryl sulphate/acetone damage compared with a mixture containing ceramides alone [76]. Berardesca at al. found that an optimized topical skin lipid mixture containing ceramide 3, cholesterol and fatty acids significantly improved multiple parameters, including pruritus, after four and eight weeks of treatment in patients with atopic dermatitis, allergic contact dermatitis or irritant contact dermatitis [77]. In a 2002 Phase 1 trial of a barrier-repair emollient composed of a 3:1:1 molar ratio of ceramides, cholesterol and free fatty acids, the authors reported significant improvements in AD disease severity, TEWL, stratum corneum integrity and skin hydration after treatment [78]. A 2008 study by Huang and Chang found that the topical application of emulsions containing one or both of ceramides 1 and 3 improved the barrier function of skin pretreated with the irritant sodium lauryl sulfate [79]. The authors postulated that ceramides 1 and 3 in combination may act synergistically to decrease TEWL and increase skin hydration [79], an effect that would have great potential in combating dry skin and reducing itch. Chang et al. reported in 2018 that daily application of a formulation containing ceramide and filaggrin, the protein that binds keratin fibers within corneocytes [80], resulted in significant improvements in skin itch, dryness, hydration, desquamation, and overall quality of life of geriatric patients [81]. TEWL improved from baseline but not significantly [81], but this is likely a result of the mechanical nature of aged skin: water content of the skin tends to be lower with age as the skin thins [82]. A 2017 study by Zirwas et al. reported that a single application of a non-prescription moisturizing test material containing ceramides 1, 3 and 6-II and 1% pramoxine hydroxide resulted in a significant reduction in itch severity after 2 min, and showed continued improvement after 8 h [83]. Application of the test material up to four times in a 24 h period for six days resulted in an improvement in itch relief comparable to 1% hydrocortisone [83]. Nearly 90% of participants reported that daily use of the test material over 6 days provided itch relief for the entire night [83].

Topical products containing pseudoceramides and ceramide precursors have also been shown to improve symptoms of AD. Pseudoceramides are synthetic constructs structurally similar to ceramides but with potential differences, such as the lack of a sphingoid base [84]. Draelos and Raymond reported that a cream containing a synthetic ceramide significantly improved skin hydration and skin assessment scores in patients with sensitive skin conditions [85]. Ceramide precursors, such as phytosphingosine, are the less complex foundational parts of the more complex ceramides. A 2013 study found that an emollient containing ceramide precursor lipids improved, among other measurements, pruritus scores in AD patients [86]. While promising, the nature of pseudoceramides and ceramide precursors may make them less efficacious in treating dry skin than ceramides. We previously reported that a topical cream containing a 3:1:1 molar ratio of ceramides (subtypes 1 and 3), cholesterol and free fatty acid (Ego Pharmaceuticals Pty Ltd, Braeside, Victoria, Australia) significantly improved skin hydration and reduced TEWL compared with placebo, with these improvements also being significantly greater than those seen with formulations containing pseudoceramides or ceramide precursors [87]. 

Ceramide-containing products can also be effective in controlling symptoms of AD other than dry skin and pruritus. A 2018 double-blind, randomized, left-right comparison study by Angelova-Fischer et al. reported that the use of an emollient containing a mixture of ceramide 3, fatty acids, glycerol and licochalcone A significantly reduced the re-occurrence of flares in mild to moderate AD that had been initially cleared by corticosteroid treatment [88]. Corticosteroid treatment was discontinued prior to inclusion in this 12-week study, and the arms treated with placebo experienced significantly worse clinical scoring of atopic dermatitis (SCORAD) and increased TEWL and itch severity [88]. The significant reduction in SCORAD is particularly noteworthy, as the test material used in this study is a non-prescription emollient that can be used indefinitely. Ma et al. reported that a skincare regimen incorporating the twice-daily application of a ceramide-containing moisturizer (and once-daily cleansing with a body wash) both significantly delayed flares and significantly decreased the overall number of flares after 12 weeks in children with a history of mild to moderate AD that had been successfully treated initially with topical corticosteroid [89]. Similar to the study in [88], corticosteroid treatment was discontinued prior to inclusion in the 12-week study. By Week 12, children receiving ceramide-containing moisturizer also had less skin dryness and burning [89]. Similarly, another 2017 study, this one by Koh and colleagues, found that twice-daily use of a ceramide-containing moisturizer for 12 weeks in children with mild to moderate AD significantly improved both carer-assessed patient eczema severity time (PEST), a measure of disease severity made by the sufferer or carer, and clinician-assessed SCORAD [90]. 

Whether ceramides proper, pseudoceramides or ceramide precursors, the well documented effects of topical ceramide products in increasing skin hydration, decreasing TEWL and improving the severity of flare ups to a degree comparable with corticosteroids make them efficacious, safe [91] and inexpensive adjunct therapies for restoring xerotic skin, but it is the ability to use these products indefinitely that makes them ideal for reducing itch and helping to manage the symptoms of AD. 

## 6. Topical Pine Tar: Itch Relief Millennia in the Making

As described above, first line treatment for AD is the use of topical corticosteroids, but only for short- to medium-term durations. Topical delivery of ingredients found naturally in the skin can help to optimize barrier performance, reducing pruritus indirectly, but is there an active ingredient that can specifically target pruritus therapeutically without the side-effects associated with long-term corticosteroid use? The most effective known ingredient is pine tar. Mass produced topical preparations containing pine tar have been available around the world for over a century, and modern over-the-counter preparations are available in various formulations to help tailor its use to the patient’s needs, including gels, lotions and bars [92]. Pine tar, the end product of the destructive distillation of pine wood in extreme temperatures, has been used for centuries for everything from the preservation of ship decking in Scandinavia to its use as a flavoring in the food industry [93]. Its use as a therapeutic agent, however, extends as far back as the age of the father of medicine himself, Hippocrates [94]. Despite this long history of use, the actual mechanism of action and therapeutic activity of pine tar is poorly understood, due simply to the fact that its chemical complexity precludes it from being standardized. As such, its proposed mechanism of action has been extrapolated from studies of coal tar, another commonly-used tar for therapeutic purposes.

What *is* known about pine tar, however, is that it displays potent anti-pruritic and anti-inflammatory properties [95], and is commonly indicated for use in relieving the itch and inflammation associated with numerous chronic itchy skin conditions, including atopic dermatitis and psoriasis [96]. The major benefit of pine tar for the management of chronic itch is its steroid sparing effect; its anti-inflammatory and antipruritic properties reduce itch and the need to scratch, helping to reduce the incidence of AD flare ups while limiting or even eliminating the need for topical corticosteroids. Langeveld-Wildschut et al. treated six patients with 10% pine tar in cetamacrogol ointment, 0.1% triamcinolonacetonide in cetamacrogol ointment or cetamacrogol ointment alone on three separate parts of the back daily for three weeks before patch testing and immunohistochemical analysis of skin biopsies [97]. Pine tar was found to have comparable inhibitory effects to the corticosteroid on the cellular constituents of allergic inflammation, including IL-4^+^ and CD1+ cells, eosinophils and T-cells [97]. A recent pilot study by Hon et al. compared the efficacy of two complementary bath products, one a pine tar solution (Ego Pharmaceuticals Pty Ltd, Braeside, Victoria, Australia) and the other a preparation containing green tea extract, in the reduction of moderate to severe AD disease severity in children [98]. Daily bathing with the pine tar solution showed significant improvements in, among other parameters, SCORAD, Patient Oriented Eczema Measure (POEM) and Children’s Dermatology Life Quality Index (CDLQI) scores after four weeks, each of which measure skin itchiness as the primary symptom [99,100]. The fact that bathing with pine tar can improve AD scoring, coupled with its steroid sparing effect, positions it as an attractive alternative to commonly used treatment strategies for AD such as bleach baths. While studies suggest that bleach baths may be beneficial in the treatment of AD, the nature of bleach baths present many issues. For one, bleach is a household chemical, so is not manufactured to the same level of quality as a therapeutic agent like pine tar. Therapeutic products are subject to strict guidelines covering safety and efficacy data, as well as the requirement to be manufactured according to Good Manufacturing Practice (GMP). Further, the use of bleach baths requires the correct concentration of bleach in water. As a household chemical, there is no standard concentration available. To compound the issue, NaOCl, the active component of bleach, degrades over time so that the concentration of bleach added to baths can vary wildly not only over time but between manufacturers and even different batches of the same product. Adding too much NaOCl to a bath risks irritation and burns for little therapeutic value [101].

The potent anti-pruritic potential of pine tar is bolstered by its safety profile; the minimal phenol content of commercially-available pine tar products means that toxicity is unlikely [96], and, unlike coal tar, pine tar does not cause photosensitization [92]. Crucially, pine tar also does not have the carcinogenic potential that is often attributed to coal tar. A study by Swallow et al. found that a commercially-available pine tar solution (Ego Pharmaceuticals Pty Ltd, Braeside, Victoria, Australia) contained no detectable levels of four of the eight polycyclic aromatic hydrocarbons (PAH) known to cause carcinogenicity in animals [102]. Only minimum detectable levels were found of the other four PAHs, up to 300-fold less than that found in the commercially-available coal tar products [102]. 

## 7. Conclusions

Chronic pruritus, and the scratching it necessitates, can have profound physical and psychological effects on sufferers. Given its complex nature, most treatment strategies for chronic itching are unsatisfactory and there is a great need for easily accessible, inexpensive topical options to help manage the urge to scratch. In this review, we provide a brief overview of the pathophysiology of pruritus, its role in atopic dermatitis, and the role the stratum corneum can play in managing the itch. We also briefly discuss the potential of two topically-delivered management options for chronic pruritus: ceramide-dominant emollients and pine tar-based preparations. As adjunct therapies for pruritic skin diseases, the efficacy of both options, especially their steroid-sparing effects, present them as cheap, safe and easily-accessible choices for patients and clinicians that can be used indefinitely without fear of adverse reactions.
